# Learning Compassion and Meditation: A Mixed-Methods Analysis of the Experience of Novice Meditators

**DOI:** 10.3389/fpsyg.2022.805718

**Published:** 2022-04-05

**Authors:** Jennifer S. Mascaro, Marianne P. Florian, Marcia J. Ash, Patricia K. Palmer, Anuja Sharma, Deanna M. Kaplan, Roman Palitsky, George Grant, Charles L. Raison

**Affiliations:** ^1^Department of Family and Preventive Medicine, Emory University School of Medicine, Atlanta, GA, United States; ^2^Department of Spiritual Health, Emory University Woodruff Health Sciences Center, Atlanta, GA, United States; ^3^Graduate Division of Religion, Emory University, Atlanta, GA, United States; ^4^Department of Behavioral, Social, and Health Education Sciences, Rollins School of Public Health, Emory University, Atlanta, GA, United States; ^5^Department of Psychiatry and Human Behavior, Brown University, Providence, RI, United States; ^6^Department of Behavioral and Social Sciences, Brown University, Providence, RI, United States

**Keywords:** compassion, contemplative, meditation, individual differences, qualitative

## Abstract

Over the last decade, numerous interventions and techniques that aim to engender, strengthen, and expand compassion have been created, proliferating an evidence base for the benefits of compassion meditation training. However, to date, little research has been conducted to examine individual variation in the learning, beliefs, practices, and subjective experiences of compassion meditation. This mixed-method study examines changes in novice meditators’ knowledge and contemplative experiences before, during, and after taking an intensive course in CBCT® (Cognitively-Based Compassion Training), a contemplative intervention that is increasingly used for both inter- and intrapersonal flourishing. The participants in this study (*n* = 40) were Christian healthcare chaplains completing a 1-year residency in Clinical Pastoral Education (CPE) who learned CBCT as part of their professional chaplaincy training curriculum. Prior to and upon completion of training, we surveyed participants to assess their beliefs about the malleability of compassion, types of engagement in compassion meditation, and perceptions of the impact of taking CBCT. We also conducted in-depth interviews with a subset of participants to gain a qualitative understanding of their subjective experiences of learning and practicing compassion meditation, a key component of CBCT. We found that participants reported increases in the extent to which they believed compassion to be malleable after studying CBCT. We also found high levels of variability of individual ways of practicing and considered the implications of this for the study of contemplative learning processes. This multi-methodological approach yielded novel insights into how compassion practice and compassion-related outcomes interrelate, insights that can inform the basic scientific understanding of the experience of learning and enacting compassion meditation as a means of strengthening compassion itself.

“Personally, I know that compassion is a *spirit* from the biblical point of view, and I’m yearning to see how compassion is manufactured *in the lab* or in the meditative class.” (Chaplain Isaac).^1^

## Introduction

For over 30 years, scientific inquiry into compassion and its potential psychological and physiological impacts has gained momentum and sophistication (Gilbert, 2020). In psychology and related disciplines, there is growing consensus that the fundamental components of compassion are (1) reflexive experience of benevolent emotions in response to suffering, coupled with (2) the desire to aid and/or support to the sufferer (Goetz et al., 2010; Halifax, 2012; Singer and Klimecki, 2014). In addition, features and experiences associated with empathy or emotional contagion are often included in cognitive and psychological models of how compassion arises (Gilbert, 2009; Goetz et al., 2010; Feldman and Kuyken, 2011; Strauss et al., 2016; Gu et al., 2017). In the past decade, numerous interventions and techniques that aim to engender, strengthen, and expand individual compassion have been formalized, attracting additional research interest in their mechanisms and efficacy (Graser and Stangier, 2018). Contemplative approaches to strengthen compassion are becoming increasingly common across experimental, clinical, and educational settings, often presented to first-time meditators from diverse educational and cultural backgrounds (Hofmann et al., 2011; Graser and Stangier, 2018; Sinclair et al., 2021a,b).

Multimodal evidence indicates that within an experimental group or population, compassion meditation interventions lead to both primary (e.g., compassion and altruism) and secondary benefits (e.g., hope and relationship satisfaction) for some people (Pace et al., 2009; Leiberg et al., 2011; Mascaro et al., 2012, 2016, 2021; Wallmark et al., 2013; Bach and Guse, 2015; Roeser and Eccles, 2015; Hildebrandt et al., 2017; Kirby, 2017; Kirby et al., 2017; Matos et al., 2017; Brito-Pons et al., 2018; Luberto et al., 2018; Ash et al., 2020; Austin et al., 2021). However, individual outcomes vary widely, and causal mechanisms remain obscure. Research examining the subjective cognitive and emotional experiences and the learning processes that occur during compassion training is in its infancy and has received less attention than studies on long-term meditators or novices learning mindfulness-based practices (Irving et al., 2014; Rappert et al., 2016; Petitmengin et al., 2017; Kordes et al., 2019).

Examining individual variation in the learning, practice, and subjective experience of compassion meditation is critical because quantifiable factors such as practitioner goals (Shapiro, 1992; Sedlmeier and Theumer, 2020) and cumulative practice time (Jazaieri et al., 2013; Jha et al., 2017; Rooks et al., 2017) have been shown to impact the effectiveness of contemplative interventions. However, the wider array of experiences and learning factors in contemplative training has received comparatively little attention (Gully et al., 2002; Caspi and Bell, 2004; Tang and Braver, 2020). It is vital that research into compassion practices gain insights into sources of individual variation in the subjective experience and process of training to facilitate discovery of why, when, where, and for whom compassion training and practice would be most effective.

Meditation in modern thought and culture has been closely associated with the goal of attaining unusual experiences and states of consciousness (Sharf, 2000; Tweed, 2005; McMahan, 2008; Payne, 2012; Harrington and Dunne, 2015). However, the familiar and mundane experiences of the mind and body (or mind–body) engaged in learning to meditate—frustration, difficulty, mind wandering, falling asleep, self-evaluation, physical discomfort—can also be a common part of learning to meditate and are relevant to novices’ learning processes (Hasenkamp et al., 2012; Lomas et al., 2015). In an introductory course, novice meditators are generally tasked with (1) becoming familiar with the skills and strategies for practicing compassion meditation, (2) learning a framework of concepts and goals in which devoting time and energy to compassion meditation makes sense, and (3) internalizing and habituating compassionate perspectives and responses through repeated practice. Indeed, some meditation novices may initially be as concerned about learning *how to meditate*—avoiding and correcting missteps, following prescribed forms, memorizing sequences of steps—as they are about learning to change their perspectives *by means of meditating*. These constitute two distinct types of learning: skill development and adaptation on the one hand and belief revision on the other (Ohlsson, 1996). Learning processes, both deliberate and effortful or implicit and automatic, represent a key domain of individual variation in contemplative training and are highly pertinent to the context of compassion meditation interventions in which instructors convey skills and encourage repeated exercises for the trainee to master (Ozawa-de Silva and Negi, 2013; Ash et al., 2021).

To examine individual variation in learning processes that occur during compassion training, the present study explores how a group of novice meditators begin learning to practice a compassion meditation method known as CBCT® (Cognitively-Based Compassion Training). Previous research indicates that CBCT increases hope (Reddy et al., 2013) and reduces symptoms of depression in healthy populations (Desbordes et al., 2014; Mascaro et al., 2016) and attenuates the pro-inflammatory response to psychosocial stress (Pace et al., 2009, 2013). In breast cancer survivors, CBCT reduced depression and psychological stress associated with the fear of cancer recurrence (Dodds et al., 2015; Gonzalez-Hernandez et al., 2018). In a pilot study of chaplain residents, CBCT was associated with decreased burnout and anxiety (Ash et al., 2020). Our first two research aims focus on two distinct forms of cognitive change that participants may undergo while learning CBCT. First, we examined changes in beliefs about compassion, given previous research suggesting these changes may be an important component to compassion training (González-Hernández et al., 2021) and may predict its effects (Schumann et al., 2014). Second, we examined the development of the techniques of CBCT meditation practice and the process of incorporating them into one’s life. In addition, our third aim (3) is to understand novice practitioners’ perceptions of whether and how compassion meditation training had been beneficial. These aims were evaluated using quantitative self-report measures and expounded with greater nuance using semi-structured interviews to uncover and characterize sources of individual variability.

## Materials and Methods

### Research Overview

The data reported here were collected as part of a larger study, approved by the Emory University Institutional Review Board, to evaluate the effects of CBCT on chaplain residents enrolled in a clinical pastoral education (CPE) program. The parent study was a longitudinal, randomized, wait-list controlled study that was preregistered (NCT03529812), with chaplain-reported depression, anxiety, burnout, and empathy as the primary outcomes, and patient-reported reductions in anxiety and depression as well as satisfaction with the chaplain-delivered spiritual care encounter as secondary outcomes. We will report the results of this larger effectiveness study in a separate manuscript. Here, we report the results of a mixed-method examination of the learning processes and subjective experience of chaplains enrolled in the parent study. The sources of data for this study include longitudinal and wait-list controlled measures to assess beliefs about the malleability of compassion with CBCT training. In addition, we collected a chaplain-reported measure about the perceived benefits of CBCT. To provide a richer context for these quantitative self-report measures, we obtained chaplains’ detailed responses to interview questions regarding their ways of enacting and personalizing CBCT meditation procedures to suit their needs and inclinations. Chaplains provided written informed consent prior to participating.

Chaplain residents were randomized to receive CBCT either in the fall or spring unit of their CPE residency year. All study participants completed a self-report measure of compassion malleability (described below) prior to and immediately upon completion of CBCT. Chaplains randomized to CBCT also completed post-CBCT self-report measures about meditation practice and perceived benefit (described below). For the qualitative interview portion of the study, we invited all chaplain residents to participate at three timepoints relative to their CBCT training: (1) prior to any training, (2) after the halfway point in the CBCT course, and (3) shortly after the end of the course. Each chaplain resident was encouraged to participate in an interview at as many time points as they were able. Chaplains were consented for each of the three interviews separately.

### Participants

During the study period, participants were completing a year-long residency of an ACPE-accredited CPE program at Emory Healthcare that recently incorporated CBCT into its educational curriculum. The inclusion criterion was enrollment in the CPE program as a hospital chaplain resident to provide spiritual care to patients in acute-care hospital settings. All chaplain residents received CBCT as part of their residency training, but participation in the research described here was voluntary. There were no exclusion criteria.

Chaplain residents learn to deliver spiritual care in CPE programs, which bring theological trainees of all faiths into supervised encounters with persons in the healthcare system who may be experiencing a crisis. Although the CPE program at Emory attracts individuals from a broad array of faiths, it skews heavily to Protestant Christian, likely the effect of several Protestant seminaries in the area from which many residents matriculate. Residents’ clinical work occurs in the context of formal and informal feedback from peers and educators, with the goal of developing trainees’ awareness of themselves, skills in interpersonal and inter-professional relationships, and appreciation of the needs of those to whom they provide care. Their responsibilities during training include responding to cardiac arrest codes, deaths, staff requests, recent admissions, and patient or family requests. Chaplain residents also assist patients in end-of-life planning and the completion of advance directives. In addition, chaplain residents address the religious and spiritual needs of hospital staff, including bereavement from death of patients as well as distress arising from events in their personal lives. Chaplain residents in the Emory program are assigned to one of five hospital locations for most of their instructional and clinical activities.

### Randomization and Blinding

Chaplain residents were randomized to receive CBCT during the fall unit of CPE or to continue standard CPE as usual (the wait-list comparison group) and receive CBCT in the spring unit. Chaplain residents were randomized by hospital location using the RANDBETWEEN function in Excel, such that within each hospital location there were a roughly equal number of chaplains randomized to the CBCT and wait-list groups. All study participants were blind to group assignment at the Time 1 assessments, and all research personnel were blind throughout the entirety of quantitative data collection, data entry, and statistical analysis.

### Cognitively-Based Compassion Training

CBCT is derived from Indo-Tibetan Buddhist mind-training, or *lojong* (Tib: བློ་སྦྱོང་, Wylie: blo sbyong), techniques that combine exercises for stabilizing attention and calming the mind with contemplation of aphorisms, visualizations, self-inquiry, and related meditative exercises for reinforcing and internalizing compassionate perspectives. CBCT, however, was adapted to be accessible to those of any or no faith tradition. The meditation exercises and reflective practices that comprise CBCT are described as a “cognitive” or “analytical” style of meditation. In contrast to compassion meditation practices that primarily focus on the somatic experiences associated with compassion (e.g., sensations of warmth and caring in one’s chest), the practices taught in CBCT emphasize critical thinking, mental investigation, and reflection, with the ultimate goal of arriving at personal insights about one’s own life, relationships, and experiences (Silva and Dodson-Lavelle, 2011; Ash et al., 2021).

The cognitive exercises and target perspectives of CBCT are organized into sequenced learning modules (Ash et al., 2021). The preliminary or foundational module trains practitioners to evoke a scene or memory that represents a “moment of nurturance,” which becomes a touchstone experience of compassion that practitioners seek to both develop in themselves and offer to others in the following modules. In modules one and two, practitioners learn and practice fundamental mindfulness skills that correspond (respectively) with focused attention (FA) on a single object—typically the breath—and open monitoring (OM) of sensory perceptions and endogenous mental phenomena as delineated by Lutz et al. (2015). In contrast, modules three through six have a more analytical focus. In module three (Self-Compassion), students bring aphorisms, examples, and personal experiences to mind while meditating to discern habits of thought and behavior that exacerbate personal stress. This is followed by engendering a firm resolve to change those self-defeating patterns and to garner patience and tolerance of stress and suffering as universal human experiences, while simultaneously recognizing the difficulty and slow pace of achieving lasting personal change.

During modules four, five, and six, practitioners use a similar analysis of personal relationships and experiences to dislodge perspectives that inhibit compassion; however, in these modules the focus is on others rather than oneself. In module four (Impartiality and Inclusivity), practitioners learn to assess and equalize the ordinarily biased attitudes toward friends, adversaries, and those to whom we feel little connection. In module five (Deepening Gratitude and Tenderness), practitioners reflect on the recognition that our wellbeing is due to the cooperation and kindness of countless other people. The resulting feeling of thankfulness then combines with the recognition that everyone is vulnerable to experiencing suffering and hardship to form a basis for feelings of endearment toward others. From this tender regard for others, practitioners move to module six (Harnessing the Power of Compassion), which begins with wishing others to be relieved from suffering and progresses to generating the motivation and the readiness to help bring that about if the opportunity arises. While progressing through the entire sequence of modules represents a prototypical CBCT meditation session for an experienced practitioner, the CBCT teachers in this study did not set an expectation that CBCT meditation sessions should always include elements from each of the learning modules, but rather encouraged chaplains to experiment and find what worked best for them.

Chaplain residents randomized to the CBCT group met weekly for four full-day meditation workshops held in a local Protestant church. The training days focused on the sequence of CBCT modules, covering on average two modules per day with their associated contemplative steps and exercises. Between training days and in the weeks following the course, residents were encouraged to practice CBCT in their free time either by listening to recorded audio of meditation guidance by a CBCT instructor or by guiding themselves through one or more of the contemplative exercises they had learned in class. Instructors provided audio recordings tailored to each of the CBCT learning modules with options varying in duration between approximately 5–30 min.

### Self-Report Measures

#### Compassion Malleability

To examine chaplain residents’ beliefs about compassion, we used a measure originally developed to examine changes in beliefs about the malleability of empathy (Schumann et al., 2014). We made two important modifications to the published measure. First, we substituted the word “compassion” for “empathy” in all items (e.g., “A person’s level of *empathy* is something very basic about them, and it cannot be changed much” became “A person’s level of *compassion* is something very basic about them, and it cannot be changed much”). Second, we added two additional items to tap into and differentiate between the affective and behavioral aspects of compassion: (1) “People can learn to think and feel more compassionately and thereby become more compassionate,” and (2) “People can learn to speak and behave more compassionately, and thereby become more compassionate.” Participants used a Likert scale to indicate their level of agreement with each statement ranging from 1 (“Strongly disagree”) to 7 (“Strongly agree”). Three items were reverse-scored. A higher score on this scale indicates a relatively greater belief that compassion is a malleable skill that can be developed. All study participants—residents randomized to CBCT and those randomized to the wait-list group—completed this measure prior to randomization. Residents randomized to the CBCT group completed the measure again immediately upon completion of CBCT, and residents randomized to the wait-list group completed it at the same time. Cronbach’s alpha calculated from the current study indicates that this adapted version of the measure had good reliability: Time 1 *α* = 0.81, Time 2 *α* = 0.87.

#### Perceived Benefits of CBCT

To characterize chaplain residents’ perceived benefits of CBCT, at the Time 2 assessment we asked chaplain residents randomized to CBCT (*n* = 21) to report their agreement with the statement, “I benefited from learning CBCT” using a Likert scale (1 = “Strongly disagree,” 7 = “Strongly agree”). Using the same Likert scale, we also asked them to report on more specific benefits, including helping to improve personal relationships, improve physical health, improve mental health, and improve their spiritual health consultations with patients.

#### Practice Time

To quantify self-reported practice time, at the Time 2 assessment we asked chaplain residents randomized to CBCT to estimate (1) how often they practiced CBCT outside of class time using the online recorded meditations and (2) how often they engaged in self-guided CBCT meditation—without the use of recordings—outside of class time. For both questions, their response choices were as follows: more than once/day, about once/day, 2–3 times/week, once/week, or virtually never.

### Statistical Methods (Quantitative)

Quantitative responses were analyzed using Statistical Package for the Social Sciences (SPSS) software (version 27.0 for Windows, SPSS, Inc., Chicago, IL, United States). Missing items in the beliefs about compassion scale were estimated with expectation maximization (Graham, 2009; missing items never accounted for more than 5% of total data) using other items within the scale as predictor variables. We used the Shapiro–Wilk test to assess data normality. All variables had a non-normal distribution, and therefore, we used nonparametric methods. To address our first aim of examining changes in beliefs about whether compassion is malleable, we used Mann–Whitney U tests to assess whether attitudes toward compassion differed between groups at baseline and at Time 2 after CBCT. We used analysis of covariance (ANCOVA) to determine whether the CBCT and wait-list groups differed in beliefs about compassion malleability at Time 2, controlling for Time 1 scores (Dimitrov and Rumrill, 2003). We used Wilcoxon signed-rank tests to assess within-group changes. To address our second aim of examining how novice practitioners develop and adapt the skills and techniques of CBCT meditation, we characterized self-reported practice time using recordings and adapted on one’s own. To address our third aim of evaluating the relationship between participants’ practice time and self-reported benefits, and changes in belief, we used Spearman’s rho correlation analyses to examine whether practice time was associated with changes in compassion beliefs (calculated as a difference score) or with perceived benefits in the CBCT group. Two-tailed alpha was set to 0.05 for significance for the ANCOVA and Wilcoxon signed-rank tests. The alpha level was Bonferroni-adjusted for multiple comparisons in our correlation analysis.

### Semi-Structured Interviews

All study participants randomly assigned to the CBCT group were invited to participate in semi-structured interviews about their experiences with the CBCT meditation course. Chaplains were interviewed during their normal work hours at one of four Emory Healthcare facilities or at the Protestant church facility where the CBCT course was conducted. Interviews lasted approximately 1 h and were scheduled in discrete time periods prior to, after the midpoint of, and after the CBCT course.

Interviews were conducted by a trained research facilitator with expertise in religious studies and CBCT practices specifically, and they followed a semi-structured interview guide. At the first interview, each participant was asked about their repertoires of religious and/or spiritual practices, as well as their personal understanding of the meaning and purpose of compassion. Halfway through the compassion meditation training, participants in the second round of interviews were asked about how they typically practice CBCT outside the meditation class and what the experience of doing so is like for them. They were then invited to join the interviewer in practicing CBCT for 8–10 min in silence, in the manner they would normally practice when alone. Immediately afterward, while the experience was still in working memory, they were asked to recount in detail how they had conducted their practice and what doing so was like for them. Following these subjective descriptions of compassion meditation, interviewees were then asked about any moments of insight and personal changes related to CBCT that they may have experienced. After completing the CBCT course, the third round of interviews was like the previous one. Participants were again asked to describe their typical solo CBCT practice, followed by a silent self-guided CBCT meditation period of 8–10 min, after which they described their procedure and experience of meditating while the memory of it was fresh. Further questions pertained to moments of learning, insight, and/or personal change throughout the CBCT course.

Each interview was audio-recorded and transcribed verbatim. The qualitative investigator (MPF) created a codebook of overlapping and emergent themes—e.g., meditation, compassion, subjective experience, etc.—to use when locating evidence for answering research questions. All interview transcripts were then coded and queried using thematic analysis and NVivo qualitative analysis software (Fereday and Muir-Cochrane, 2006).

## Results

### Participant Characteristics

The social and demographic characteristics of chaplain residents (*N* = 40; CBCT: 21, wait-list: 19) are presented in Table 1. Most residents (50%) were African-American or Black, and 73% had resided in the United States their entire life. The majority (73%) reported having previous experience with meditation, although none of the participants had learned or practiced CBCT. Study participants came from a broad range of Protestant Christian backgrounds. A convenience sample of 12 residents volunteered to be interviewed at least once (10 for the first interview, 11 for the second interview, 10 for the third interview), eight of whom were African-American, eight of whom were women. Four interviewees grew up outside the United States—in West Africa, in East Asia, and in the Caribbean.

**Table 1:** Sociodemographic characteristics of the chaplain residents.

		CBCT (*n* = 21)		Wait-list (*n* = 19)		*P*
		*N*	%	*N*	%	
**Gender**	Female	9	43	13	68	0.125
	Male	12	57	6	32	
**Race**	Asian	4	19	2	11	0.691
	African-American/Black	12	57	8	42	
	Afro-Caribbean	1	5	0	0	
	White	4	19	6	32	
	Missing/Unknown	0	0	3	16	
**Length of time resided in the United States**	Entire life	13	62	16	84	0.150
	11+ years	3	14	2	11	
	5–10 years	3	14	0	0	
	1–4 years	2	10	0	0	
	Unknown	0	0	1	5	
**Relationship**	Single	7	33	9	47	0.403
	Divorced	3	14	1	5	
	Single, living with someone	11	52	7	37	
	In a relationship	0	0	1	5	
	Missing/Unknown	0	0	1	5	
**Previous experience with meditation**	Yes	15	71	14	74	0.651
	No	6	29	4	21	

### Compassion Beliefs

**Figure 1:**
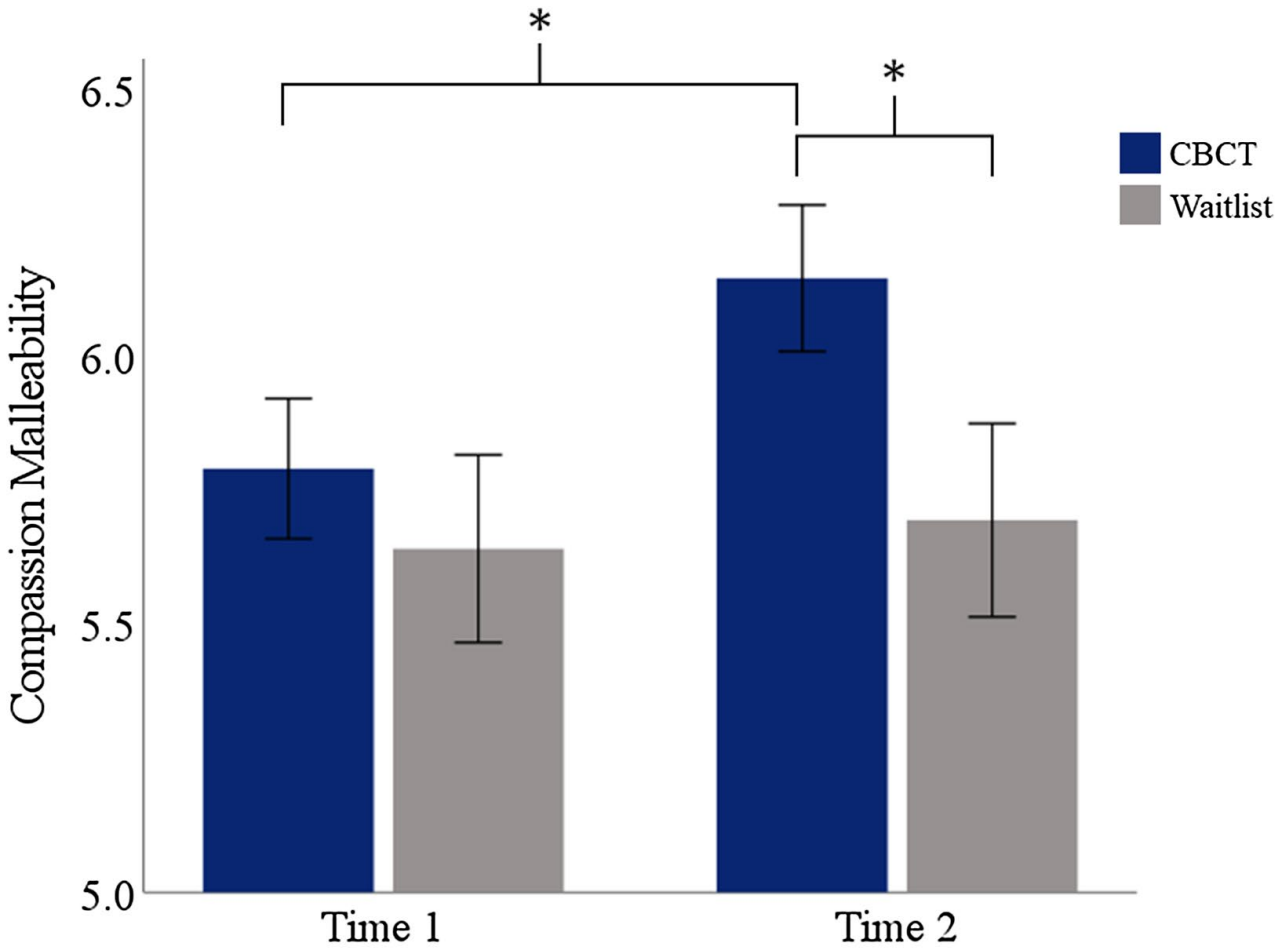
Changes in self-reported beliefs about the malleability of compassion. Asterisks indicate a statistically significant effect of group at Time 2 and a statistically significant effect of time in the residents randomized to CBCT. Error bars indicate the SEM.

Scores on the compassion malleability belief scale at Times 1 and 2 are shown in Figure 1. There was not a significant difference between the CBCT and wait-list groups in compassion malleability belief at Time 2 while adjusting for Time 1 scores. However, effects were in the expected direction, indicating that residents randomized to the CBCT group may have experienced increases in compassion malleability compared to residents randomized to the wait-list group [*F*(2, 36) = 3.76, *p* = 0.06]. Mann–Whitney U tests indicated that there was not a significant baseline difference in beliefs about compassion malleability between the CBCT group (*M* = 5.79, SD = 0.60; Mdn: 6.00) and wait-list group (*M* = 5.64, SD = 0.74, Mdn: 5.81) at Time 1 (*U* = 0.57, *p* = 0.587, *r* = 0.09). However, at Time 2 there was a significant difference in beliefs about compassion malleability between the CBCT group (*M* = 6.15, SD = 0.63, Mdn: 6.25) and wait-list groups (*M* = 5.69, SD = 0.74, Mdn: 5.88) (*U* = 2.08, *p* = 0.037, *r* = 0.33). There was a main effect of time for the CBCT group (*Z* = 2.60, *p* = 0.009, *r* = 0.57), but not for the wait-list group (*Z* = −0.13, *p* = 0.90, *r* = 0.03), indicating that the CBCT group but not the wait-list group increased the extent to which they believed compassion to be malleable. The partial Eta Squared value indicates that the effect size was small (0.10).

During the interviews, we queried residents’ beliefs about the meaning and the purpose of compassion and how CBCT impacted their beliefs about compassion. Prior to CBCT, most of the residents emphasized that compassion establishes or enhances the connection and equality between people or between a person and God, especially when a person is suffering. Some indicated that the connection depends on understanding and identifying with or simply remaining near someone who is suffering, as well as through love, forgiveness, and abstaining from judgment. Modes of helping, caring, and showing concern were also commonly mentioned as important. One response focused on compassion’s ability to purify, while another response defined compassion in non-naturalistic terms as a “spirit, from the biblical point of view,” highlighting the contrast between a notion of compassion as God-given and predetermined with a model of compassion as a malleable trait that can be deliberately engendered or developed. After CBCT, participants reported that they felt they could become more compassionate by becoming more attuned and aware of others and through practice and effort. A common theme was the importance of self-compassion for extending compassion to others. Table 2 contains a selection of excerpts from transcribed responses to these questions.

**Table 2:** Participants’ beliefs about compassion.

Theme	Representative quote
You can increase compassion	I think you can become more attuned. You become more aware. You become more compassionate, and I think most importantly, you become more present. (Vanessa)
Who you are compassionate toward can expand	I realized that I lack self-compassion, and compassion to my family, especially my dad, about what happened when I was young. And when I reflected about compassion, at first, I could not be compassionate [about] what happened in the past. And then, I kept reflecting, and then, we talked about impartiality. And then, that helped me to broaden my capacity and expand. (Nicole)
You can increase self-compassion	When doing meditation, you [can] feel like you are doing it wrong. I think being gentle with oneself during meditation is a skill—something that you learn from practice. I think that I’m learning that. (Eric)
Practice leads to improvement	There was a quote that resonates with me. I do not know where it came from, but it says, “I cannot think myself into right action, I have to act myself into right thinking.” I feel like emotions fall into those kinds of categories in many ways. … I have to continue to practice this compassion with myself in order to continue to do well at it with others. (Eric)

### Developing and Adapting the Techniques of CBCT Meditation Practice

**Figure 2:**
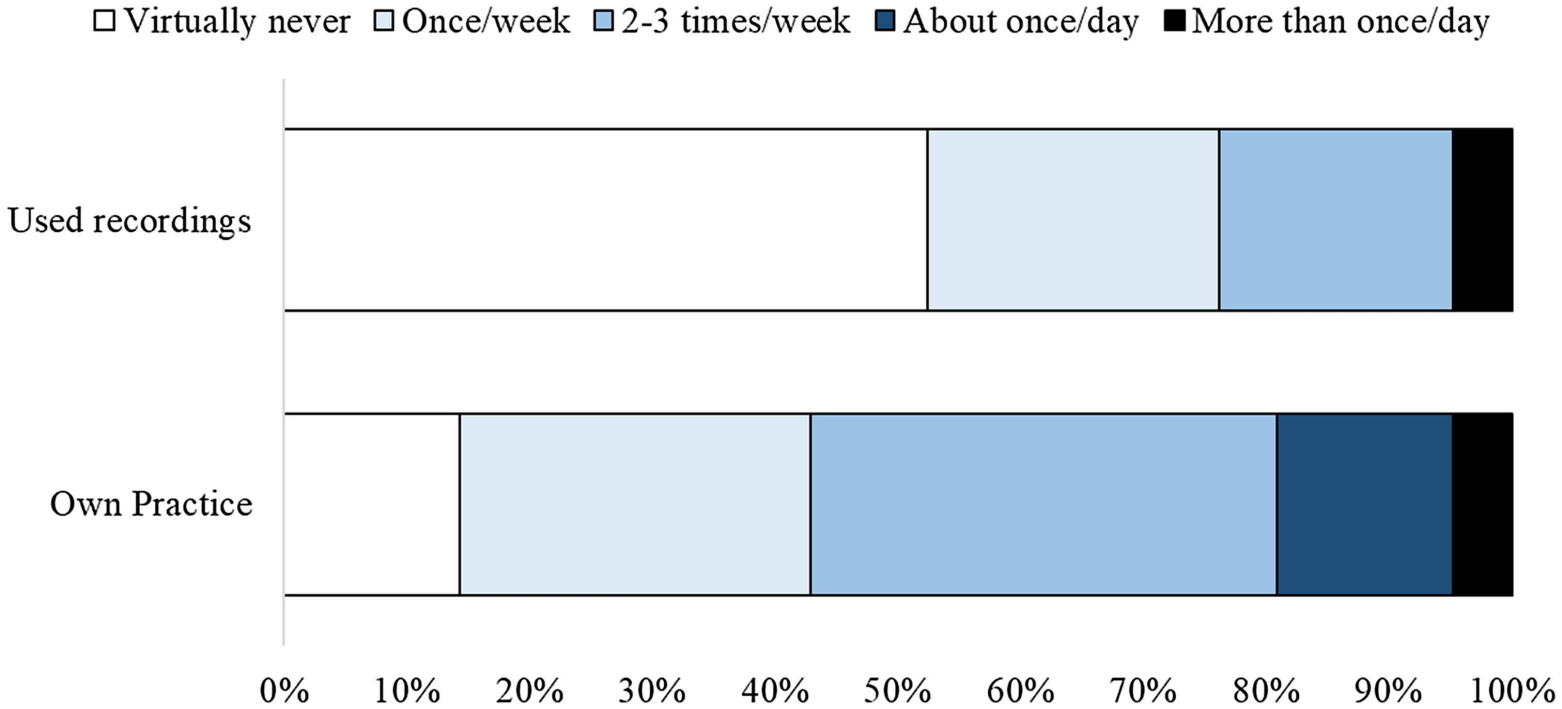
Self-reported practice time depicted according to the frequency of practice on their own and using guided audio recordings.

Regarding our second research aim of examining the development of meditation skills, we characterized chaplains’ frequency of CBCT practice both using the accompanying audio-recorded meditation guidance and without using the recordings (Figure 2). We found that, of the 21 chaplain residents randomized to the CBCT group, 86% (18/21) reported practicing CBCT one or more times per week outside the meditation class *without* listening to audio-recorded meditation guidance. Thirty-eight percent (8/21) of participants practiced in this way 2–3 times per week, almost 20% (4/21) did so once per day, and 14% (3/21) virtually never engaged in self-guided practice (i.e., without using audio-recorded guidance) outside of the meditation class. In contrast, most chaplains (52%; 11/21) reported that they virtually never listened to the recordings when practicing CBCT. The remaining 48% (10/21) of participants reported using the audio recordings at least weekly, of which half (24%) reported that they used the recordings two or more times/week.

In our interviews to understand what chaplain residents reported experiencing and doing during meditation, residents reported incorporating CBCT practice into their daily work and household routines, sometimes blending elements of compassion meditation with everyday tasks, such as driving to work or walking between hospital buildings (Table 3). They also commonly reported blending CBCT practice in with familiar religious and spiritual activities, such as prayer and scripture study. While meditating, most interviewees described drawing upon the earliest contemplative exercises of resting in a moment of nurturance and focusing their attention on a neutral object as the most easily accessible resources to bring to mind when practicing CBCT in a spontaneous moment. Developing their knowledge of these and later analytical and compassion-specific contemplative exercises brought challenges and obstacles for residents to overcome and/or accept, described in more detail below.

**Table 3:** Developing and adapting skills and techniques.

Theme	Representative quote
CBCT aligns with current practice	I’ve been doing this, just not realizing that this is what I’ve been doing, you know, in some fashion, maybe not ‘to the letter,’ or ‘to a T’ …and so, that’s what I mean when I say that it is comforting to know ‘Oh, *this* is what I’ve been doing,’ to have a name to go with it… (Danielle)
Engaging spiritual practice through the lens of CBCT	[CBCT is] even helping me now to be able to hear the voice of the Lord—personally. And, when I read the Bible, as a believer, as Christian, it has come alive to me. … It is making sense to me. Just this morning, after everything, when I got into my car. I took my phone—I have this Bible gate something. I was warming the car. I just wanted to know the verse of the day and they led me to the Bible verse: Jeremiah Chapter 29, Verse 11. And it said, “I have thoughts and plans for you, says the Lord. Thoughts of welfare and not harm.” And so, it gives me cause to just reflect on it, just for a moment. Those words were given some meaning. It gave me reassurance and energy for the day. … God wishing me well and wishing them well—a sense of endearment. God is so endeared to me and to them. In spite their many sufferings, in spite of all their difficulties. This is God’s thoughts about me. It does not mean that problems will not be there. It does not mean that pain will not be there. It does not mean that sickness will not be there. But, in their midst of all that, God’s heart is so much to me. That gives me a kind of resilience. And even when I’m out of the zone [of resilience], this reassures me, and I come back. (Isaac)
Skills in letting thoughts arise and pass	The thoughts might not change, but at least I’m prioritizing them. As to what will bring me endearment, compassion, generate energy in me, and those thoughts that might be harmful: just as they are sparks, just try to push them, just let them go. It’s just like a sieve—to filtrate. Let the harmful ones go, and the good ones that will help with my endearment, to generate some warm wholesome energy in me that I will also be able to give that to others. (Isaac)

#### Ways of Practicing CBCT

During in-depth interviews about CBCT practice, study participants reported engaging with CBCT meditation by adapting and individualizing their own procedures for practice. The training course presented a prototypical mode of CBCT practice in which the practitioner goes through a series of contemplative exercises over a sustained period of time, usually between 5 and 25 min. The exercises include visualizations, perspective-taking, introspective questioning, recollecting compassion-conducive aphorisms, and strengthening emotional and motivational states that propel compassion. Participants had the option to meditate while listening to audio-recorded meditation guidance similar to the verbal guidance provided by the instructor in class, or they could practice without outside guidance. Individuals who practiced without the use of audio recordings tended to adapt and individualize their own procedures for CBCT practice, including blending it with other spiritual and religious practices in their repertoires, as discussed below. Not all participants reported engaging with CBCT in the prototypical way, remaining still and seated. A small number of residents reported finding this type of meditation unpleasant and/or awkward and only engaged in it while in the CBCT class. Several others tried to engage in still and seated meditation initially before deciding it was not conducive for them. Still others reported that they engaged more with seated and still meditation for longer periods of time as their knowledge and experience of CBCT increased and deepened over time.

Nearly all the residents who participated in the interviews also reported brief, intermittent engagement with CBCT by pausing briefly during their daily activities to bring to mind either a moment of nurturance or to breathe mindfully. They reported that the purpose of doing so is to momentarily attune to a personal intention and purpose, a spiritual or religious identity, an existential framework, such as being in God’s care, or with the orientations and perspectives that facilitate compassion, according to CBCT. Interviewees reported that this momentary attunement might last anywhere from a few seconds to several minutes, that it is extemporaneous and imperceptible to others, and that it does not involve questioning and analysis. In addition to momentary yet deliberate CBCT practice, interviewees also reported inadvertent engagement with CBCT as its perspectives and aphorisms naturally came to mind in the midst of activities and interactions to which they were relevant.

#### Practice Blending

Most chaplains interviewed described a type of practice blending, which involves mixing CBCT with their preexisting spiritual and religious practices and consciously invoking their religious commitments and understandings during meditation practice. For example, some chaplains incorporated a period of meditation during part of the time that they usually set aside for religious devotion and prayer at the beginning of the day. Interestingly, residents also used exercises and strategies from CBCT to augment religious and spiritual practices such as scripture study and prayer, for example by using the mental stabilization strategies to help them prevent mind wandering during both study and prayer. Practice blending tended to develop over time as participants’ familiarity with CBCT increased. In addition to practice blending, chaplains enthusiastically connected various concepts and ethics that they learned in the CBCT course to parallel practices and teachings from their religious traditions within Protestant Christianity, and especially ones associated with stories and quotations from the Bible.

#### Learning Obstacles

Residents reported three distinct obstacles to practicing CBCT to their satisfaction. First, several reported “nodding off” (falling asleep) while meditating at home and during guided meditation in the CBCT classes. In addition, many reported lacking *opportunity* to practice on their own time and gain greater familiarity with the different facets of CBCT. They needed more space, privacy, quiet, and/or free time than they could find either at home, at the hospital, or both. A small number of residents reported initial concerns about incompatibility between CBCT and their religious traditions. All participants were Protestant Christians from a wide spectrum of denominations, each with clerical and theological backgrounds. While most saw no conflict between CBCT and their religious faith, some reported that they engaged with CBCT meditation *despite* believing that their co-religionists would object to it, and others reported that they declined to participate in meditation at all in the initial stages of CBCT training. However, these residents reported that their reservations did not persist past the second training session. One chaplain who had religious misgivings and said prayers while her peers were trying out CBCT meditation for the first time reported that she resolved her concern that meditation would transgress her faith commitments by redefining CBCT as self-care and not especially relevant to her religious identity. Others reported finding strong parallels between their religious teachings and the concepts presented in CBCT, allowing them to see the practice of CBCT as in alignment with their religious identity.

In addition, many chaplains reported relying heavily on the early CBCT modules: resting in a moment of nurturance, focusing attention on an object, and monitoring the flow of mental experience and were hesitant to progress past those steps in the protocol. While any given CBCT meditation session might not cover all the learning modules, both in the CBCT manual and in the audio-recorded meditation guidance, the concepts and contemplative exercises from the two earliest learning modules are most likely to be included at the beginning of meditation sessions, even when working on one of the later analytical modules. This suggests that the earlier modules may have felt more accessible than the later modules because they are more heavily practiced, providing participants with greater opportunity for familiarity and comfort. Several chaplains were apprehensive that combining all the modules together would make CBCT (a) too difficult to master, or (b) too cumbersome to be practiced regularly.

#### Awareness of Knowledge Learned

Several chaplains reported difficulty remembering the entire CBCT sequence outside of the group training context. However, despite reporting difficulties internalizing and incorporating the latter modules of CBCT, when participants were asked to describe a self-guided CBCT meditation session that they had just completed as a component of the second and third rounds of interviews, several participants were able to recall more elements from the latter analytical CBCT modules than their description of learning difficulties seemed to suggest. In addition, descriptions of subjective meditation experiences and sequences of contemplative steps and strategies differed depending on whether the respondents were recalling a meditation session they had just completed and that was still fresh in their working memory or whether they were recalling ways of practicing CBCT based on experiences of meditating the previous day or during the previous week. Recollections of having just finished meditating were much more detailed, afforded more content with which to draw connections and comparisons with the course material, and covered more of the CBCT modules.

### Beliefs About Effectiveness of CBCT

**Figure 3:**
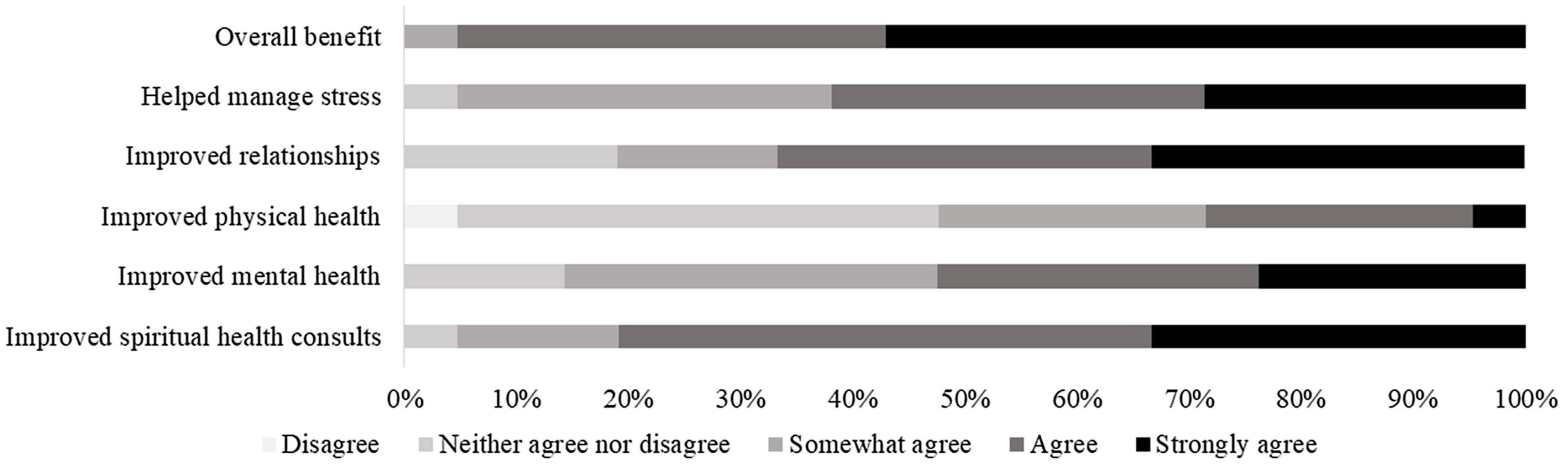
Self-reported benefits of CBCT. Of note, no participants endorsed the “somewhat disagree” or “strongly disagree” options.

A measure of chaplains’ perceptions of the effectiveness of learning and practicing CBCT showed that 95% of respondents agreed or strongly agreed that they benefited from learning CBCT. In addition, a majority (81%) agreed or strongly agreed that CBCT improved their spiritual health consultations in the hospital. Sixty-seven percent agreed or strongly agreed that CBCT improved their personal relationships, 52% agreed or strongly agreed that CBCT improved their mental health, and 29% agreed or strongly agreed that CBCT improved their physical health (Figure 3).

There was a positive association between the frequency of CBCT practice and perceptions at Time 2 that the CBCT course was beneficial [*r_s_*(21) = 0.57, *p* = 0.007], that CBCT would improve their spiritual health consultations [*r_s_*(21) = 0.49, *p* = 0.025], that CBCT improved their personal relationships [*r_s_*(21) = 0.64, *p* = 0.002], and that CBCT improved their mental health [*r_s_*(21) = 0.53, *p* = 0.014]. After Bonferroni adjustment to account for multiple comparisons, meditation practice remains significantly associated with the perception that CBCT improved participants’ personal relationships at an alpha level of 0.005. There was a positive association between changes in beliefs about compassion and perceptions at Time 2 that CBCT improved mental [*r_s_*(21) = 0.52, *p* = 0.015] and physical [*r_s_*(21) = 0.45, *p* = 0.039] health (Table 4). In interviews, residents reported that CBCT was effective in increasing self-compassion and for improving their ability to identify and understand their emotions. One resident reported the belief that CBCT will help make compassion her “second nature,” making her compassion more effortless and effective. Another chaplain reported having several “ah ha” moments during training. For example, early in training, while engaging with the resting in a moment of nurturance module, she reported that she felt nurtured and protected for the first time. Two residents reported that they did not feel profoundly changed by their practice, though upon probing they both noted that CBCT had improved their ability to attune to their emotions and both expressed optimism that CBCT would bring about positive changes (Table 5).

**Table 4:** Correlation table of meditation practice time, changes in beliefs about compassion malleability, and perceived benefits of CBCT.

	Changes in compassion beliefs	“I benefited from learning CBCT”	“Learning CBCT will improve my spiritual health consults with patients”	“Learning CBCT improved my personal relationships”	“Learning CBCT improved my mental health”	“Learning CBCT improved my physical health”
Total meditation practice	0.26	0.57^**^	0.49^*^	0.64^***^	0.53^*^	0.16
Changes in compassion beliefs		0.24	0.24	0.37	0.52^*^	0.45^*^

* *Correlation is significant at the 0.05 level (2-tailed)*.

** *Correlation is significant at the 0.01 level (2-tailed)*.

*** *Correlation is significant at the Bonferroni-adjusted alpha levels of 0.005 (0.05/11)*.

**Table 5:** Perceptions of whether and how CBCT was beneficial.

Theme	Representative quote
“Ah ha” moment	I think, the first day of CBCT training. When we were meditating, they told us to “look at the face of the person that has nurtured you… what did you see, what did you feel?” I can say that I really, really was into the training because my “Ah ha! moment” was that I had never felt nurtured before. I have never felt protected. I have never felt that. And so, when I began to look at a person’s face and think about the things that I saw, then it’s like the light bulb came on: “Wow, my husband do love me!” And, it has just been carrying on from that moment on. I think that’s the only time I can say that I felt nurtured or felt protected is since I’ve been married with my husband. (Kim)
Emotion recognition	I learned that sometimes I felt something, and I did not realize why. And then [it would] just pass. But in CBCT, when I feel something, I kind of track [it] down, and then find the root, and kind of heal it. Not completely changing the past. It cannot change the past, but it can change my perspective or views, my interpretation. And then, actually, it liberated me. In that way, it’s very beneficial. (Nicole)
Self-compassion	It started from self-compassion. Sometimes I cannot forgive myself [for] my mistakes, or my differences in the workplace. I look… less professional, and then I’m kind of blaming myself and then beating [myself] up. And then, I shoot a lot of second arrows… toward me. So, self-compassion that I’m not perfect, but it’s fine. And that kind of relieved my anxiety level. (Nicole)
Compassion	One of the things that I know I was talking about is my compassion toward others has heightened greatly. Because now… where I used to see people from a certain lens, I’m learning how to view people from the lens of compassion, and warm heartedness. (Kim)
Recognizing common humanity	Have you ever heard someone say, “Some people in the world are not bad… everybody in the world is not bad!”? Well, what I’ve found out is, it’s not so much that everyone in the world is not bad. It’s just that everyone in the world has something that they are dealing with. And how they chose to deal with it may not be the way I would deal with it. So, it may appear that that person is bad or mean or inconsiderate or insensitive. But the truth of the matter is some people just deal with issues in life a little bit different than others. And I’m learning to see that. I’m learning to identify with those people who may handle or address things a little bit differently than I do, or than I would. I’m learning how to accept and respect the differences. (Kim)
Consistent access to compassion	When I’m doing [chaplaincy consultations] sometimes what’ll creep in is the thought that all humans want wellbeing and not suffering. And that really changes [things] when you remind yourself of that. Especially when you are about to go into a code: “I should not only be compassionate but remember that even though this is my job and I’ve seen multiple codes, for this human, this family member about to engage in this moment, this is probably one of the worst things happening to them.” And that has made my heart so much more tender going in… It really has been a profound thing for me. It does not matter if this is my twenty-fifth code, this person is in a unique setting, even if I’m used to this. And that’s really changed the way I act. (Vanessa)

## Discussion

Gaining a more detailed understanding of the effectiveness of compassion training requires researchers to identify variables that have not yet been considered and that may explain individual differences in outcomes and experiences. To aid in this effort, the current study reports findings related to processes of change, learning, challenge, and adaptation that occur when novices begin a compassion meditation program. Responses to the measure of belief in compassion malleability indicated that chaplain residents randomly assigned to CBCT showed significant change in their beliefs about the malleability of compassion. In our interviews, residents described how CBCT impacted their ability to cultivate and maintain compassion and warm-heartedness. Together, these findings are consistent with another recent study that found that CBCT training among breast cancer survivors resulted in altered semantic construction of the definition and concepts around compassion (González-Hernández et al., 2021), further highlighting the importance of changes in semantic knowledge and beliefs that occur during this analytical compassion-based intervention. There are several potential sources of increased compassion malleability belief. For one, CBCT content includes a discussion of the definition and nature of compassion, and it explicitly teaches that meditators can increase their compassion by practicing this style of meditation. Malleability beliefs may also be reinforced should participants observe an increase in their own or others’ compassionate responses to suffering, which some residents reported during the interviews.

Previous research has found that beliefs about the malleability of empathy predict empathy and empathic behavior, especially in contexts where maintaining empathy is challenging or effortful (Schumann et al., 2014). The same original series of studies examining the scale also found that those with a more malleable theory of empathy worked harder to improve their empathic accuracy as measured by the Reading the Mind in the Eyes task, suggesting that their beliefs about empathy influenced their willingness to pursue growth. This line of research emerges from a larger theoretical framework that links growth mindsets, as opposed to fixed mindsets, with positive psychological outcomes (Dweck and Leggett, 1988; Yeager and Dweck, 2020). Another line of scholarship highlights the importance of motivation for change for the success of compassion-based interventions (Steindl et al., 2018). Our findings are consistent with this, indicating that CBCT increases the extent to which practitioners believe that compassion growth is possible, which may be critical for the motivation for change. Interestingly, baseline scores on the belief in compassion malleability were relatively high and our sample contained less variance than was reported in the parent study of theories about empathy malleability (Schumann et al., 2014). This is not surprising, given that the sample in the current study was highly religious and training to be hospital chaplains. It is noteworthy that we were able to detect an effect of CBCT on beliefs even with the possibility of a ceiling effect. Although the change in scores among those randomized to CBCT was less than a point (on a seven-point scale) and the effect size was small, the effect was comparable to that of an intervention designed to alter beliefs about empathy (Weisz et al., 2021). While we cannot definitively link changes in beliefs about compassion to any positive outcomes of CBCT within this analysis, changes in belief were positively associated with perceptions that CBCT improved mental and physical health. These data indicate that compassion meditation may impart its effects in part by influencing this belief, and future research can further test this hypothesis. If such a hypothesis is supported, it would be consistent with recent observations that an unstated mechanism of effect in many psychotherapeutic interventions is the patient’s adoption of beliefs congruent with the therapy’s mental models (Goodman, 2016).

This finding is also consistent with what we know about the *lojong* tradition, from which CBCT emerged. The term *lojong* describes a process of mind training that aims to transform harmful thoughts, emotions, and behaviors into those that are beneficial to oneself and others (Silva and Dodson-Lavelle, 2011). As Geshe Thupten Jinpa writes: “whereby a process of training, habituation, cultivation, and cleansing induces a profound transformation—a kind of metanoesis—from the ordinary deluded state, whose modus operandi is self-centeredness, to a fundamentally changed perspective of enlightened other-centeredness (Jinpa et al., 2014),” CBCT was adapted from this Indo-Tibetan Buddhist tradition, and accordingly it has important differences from similar but non-analytical approaches such as lovingkindness (*metta*) meditation. While lovingkindness meditation typically involves the generation of an affective state (love and affection) that is extended outwardly to encompass ever-broadening circles of individuals, it does not typically seek to directly address cognitions that underlie hostility, prejudgment, self-focus, or bias. In contrast, CBCT focuses on teaching concepts such as interdependence and gratitude, using meditation practices and metacognitive reflective exercises to connect these concepts to personal insights. Compassionate affective states are regarded as a valuable downstream consequence of cognitive changes, but these affective states are not the primary focus. Ultimately, CBCT aims to cultivate cognitive changes that lead to an encompassing and powerful orientation of love and compassion for others. Our finding that chaplain residents report changed mindsets about the nature of compassion is in line with an understanding of how lojong and CBCT lead to meaningful change. A fruitful line of research will be to examine whether other non-analytical practices lead to similar changes in beliefs about compassion.

Chaplains’ responses during qualitative interviews reflect the manifold ways that they, as novice CBCT meditators, enacted CBCT practice for themselves. Understanding variation in how individuals actually *do* CBCT itself is a somewhat narrow question within the larger domain of experiences associated with meditation. Yet, if our science proceeds according to an implicit reckoning that the participants’ practice is a large part of what makes a contemplative intervention effective, then how they do so becomes a crucial link in the causal chain leading to individual differences in outcomes. This information reflects both what the novice meditator has done or experienced and their practical knowledge of the task, that is, the internal model of doing the action of compassion meditation that governs and drives how they enact it in different instances. Here, we found that participants relied more on practice that was unguided by the CBCT audio recordings. Interviews further revealed that residents commonly practiced resting in a moment of nurturance, a relational practice that has recently been highlighted as an often-neglected component of contemplative traditions that may be vital for helping practitioners overcome barriers to compassion and compassion training (Condon and Makransky, 2020). Our findings are consistent with this notion, given that chaplains in our study reported that this relational activity was among the more heavily used and effective components of CBCT.

Overall, the findings from this study have relevance for understanding meditation practice among novices for at least three reasons. First, a key finding that we wish to emphasize is that chaplains reported blending their spiritual and/or religious practices with CBCT meditation. CBCT instructions do not include representations of suprahuman beings, so the inclusion or importation of religious understandings and ways of relating to God or other divine personalities is noteworthy, though not unexpected. CBCT is presented as compatible with any (or no) religious beliefs, and practitioners are not asked to set aside their personal commitments to practice this meditation protocol. However, while CBCT removes religious symbols found in *lojong* (e.g., discussion of religious figures and related religious terminology), the goal of compatibility with many different beliefs and worldviews may be overly optimistic and may not adequately account for elements of *lojong* tradition that are implicit in the concepts taught in CBCT. It is relevant that responses to religious associations of *lojong* ranged from compartmentalizing, through mixing, and even rejection. Culture-mixing models have described how the mixing of religious components in a contemplative practice may depend on participants’ faith characteristics, such that participants who see the religious components as interacting with sacred values would be more likely to reject the intervention (Palitsky and Kaplan, 2021). As such models would predict, chaplains who initially rejected the practice on the basis of faith were able to adopt it after re-evaluating it as instrumental (self-care) rather than spiritual. Evidence suggests that participants who initially reject contemplative practices for religious reasons may become more amenable to engaging with them after further exposure (Mascaro et al., 2021). Our finding suggests that positive attitudes toward the intervention and opportunities to re-evaluate it as instrumental may support this process. Moreover, a portrayal of the intervention as secular may also obscure or underestimate (dis)connections between the tenets and practices of CBCT and those that are core to other religious beliefs, which may influence how CBCT is practiced and understood. This has important bearings on the common portrayal of contemplative interventions as secular, especially for practitioners who come to the intervention with a rich set of beliefs, rituals, and practices from a defined religion or spirituality. Providing participants with opportunities to autonomously reflect on connections and disjunctions between concepts taught in CBCT and their own religious commitments may be important for the acceptability of CBCT, as well as other contemplative interventions.

Second, our findings highlight the potential fallibility of recollection in the self-report of meditation practice. That is, chaplains regularly recalled implementing more steps from the CBCT protocol (especially the later more analytical modules) when they were queried immediately after meditating than they did when describing their typical CBCT meditation sessions, and some residents were genuinely surprised by how many of the modules they were able to complete without guidance from an instructor or a recorded voice. It is not clear why chaplain reports differed in this way. Behavioral recall is notoriously fraught and subject to cognitive and social bias as well as error in autobiographical memory (Schwarz and Oyserman, 2001; Parry et al., 2021). One possibility is that their memory of a “typical” CBCT meditation was an abstraction influenced by their notions of typical meditation as more in line with non-analytical mindfulness practices. However, it is also possible that the two methods for measuring and evaluating meditation practice capture distinct constructs. That is, perhaps the more immediate measurement captures actual practice, whereas the recall method captures the practice that was most meaningful or salient to the chaplains. It is also worth noting that most chaplains did not rely on audio recordings, potentially increasing their reliance on abstracted notions of the practices in declarative recall. An important implication of this finding is that prompting participants to describe their typical practice as a method to assess their level of learning and skill (i.e., a memory-based assessment) might underestimate the knowledge that participants acquired—and, therefore, the success of the protocol—compared to inviting them to discuss their practice immediately after engaging with it (i.e., a practice-based assessment).

Third, the most reported form of practice reported in the interviews was extemporaneous “off-the-cushion” practice incorporated into the activities of daily life. While we are hardly the first to point to the importance of such scaffolded practice (Lyddy et al., 2016; Vago et al., 2019), our findings are to our knowledge the first interventional study to highlight how important this component of compassion meditation may be. Here, we found that total practice time was associated with several self-reported benefits of CBCT, including overall benefits, improved spiritual health consultations, improved personal relationships, and improved mental health. Future fine-grained research should evaluate the effectiveness of CBCT practice incorporated into daily life as a mechanism of change.

Investigating the process of learning in meditation training is crucial because many participants in such interventions are meditation novices who are tasked with engendering contemplative target states—compassion, loving kindness, mindfulness—but who must first learn the basic conceptual framework in which enacting the meditation techniques and building proficiency in the techniques themselves makes sense. CBCT presents a manualized set of techniques in its series of learning modules (the foundational module plus modules 1–6). Trainees must interpret descriptive instructions both prior to and during the act of meditating. Yet, while the cognitive representations that a novice might internalize of how to enact CBCT practice are expected to correspond with the standard instructions, our findings indicate that practice was highly variable across individuals and depended on personal experience and knowledge, day-to-day opportunities, constraints, needs, and a range of other contingencies. What is more, we found that practice was important inasmuch as it was associated with perceived benefits. Self-reported practice time was positively correlated with overall benefits from CBCT, and with improvements in spiritual health consultations, personal relationships, and mental health. Prior research examining the impact of practice time on later benefits from CBCT has been mixed. In the first study of CBCT, college students randomized to CBCT who practiced, but not those who did not, had reduced inflammatory response to a laboratory stressor (Pace et al., 2010). In a study examining the impact of CBCT among adolescents in foster care, practice time was associated with decreases in C-reactive protein (CRP), an indicator of inflammation (Pace et al., 2013). In contrast, a study examining the impacts of CBCT on the wellbeing of medical students found no relationship between practice time and CBCT efficacy (Mascaro et al., 2016). The current study builds on this literature by highlighting significant variability in how participants practice as well as how they may conceive of what constitutes meditation practice (e.g., informal practice in daily life). As such, future research should consider innovative approaches to measuring practice time—and the variety of ways in which participants may elect to practice.

Limitations and future directions: the learning experiences of this group of chaplain residents are especially germane for understanding what meditation training is like for people with strong religious commitments and especially for professional religious leaders and pastoral workers from Protestant Christian religious traditions. Their learning experiences suggest sub-processes, themes, and mechanisms leading to different individual outcomes of contemplative interventions and meditation in general. While this new knowledge is important for understanding how novice meditators with active religious orientations may encounter a contemplative practice, it also potentially limits the generalizability of our findings. Hospital chaplains work in religiously pluralistic settings and are often adept at reframing and refashioning prayers, rituals, and other practices to contain combinations and abbreviations, and in the clinical context these adaptations may then be imbued with new and poignant meanings (Liefbroer et al., 2017; Liefbroer and Nagel, 2021; Mealer, 2021). All of this is crucial to a hospital chaplain’s clinical role. Our finding that chaplains creatively incorporated CBCT into their corpus of spiritual practices is among the most important findings of the current study, but future research will be important to examine how common this blending is among other novice meditators, especially those with a strong faith orientation.

## Data Availability Statement

The raw data supporting the conclusions of this article will be made available by the authors, without undue reservation.

## Ethics Statement

The studies involving human participants were reviewed and approved by Emory University Institutional Review Board. The patients/participants provided their written informed consent to participate in this study.

## Author Contributions

JM conceived of the study, oversaw quantitative data collection and analysis, helped with qualitative interpretation, and helped write the paper. MF conducted interviews, coded the qualitative data, and helped write the paper. MA helped design the study, helped interpret the findings, and helped write the paper. PP helped design the study, helped collect quantitative data, helped with data analysis and interpretation, and helped write the paper. AS helped analyze the data. DK contributed to revised drafts and provided content and expertise on the interpretation of LIWC 2015 variables. RP contributed to revised drafts and provided expertise on religion and mindfulness-based interventions. GG and CR helped conceive of the study, helped interpret the data, and helped write the paper. All authors contributed to the article and approved the submitted version.

## Funding

This research was supported by a 2017 PEACE grant from the Mind and Life Institute. Preparation of this manuscript was additionally supported by the National Institutes of Health under grants 5K01AT010488 (JM) and 1F32HL154751 (DK).

## Conflict of Interest

The authors declare that the research was conducted in the absence of any commercial or financial relationships that could be construed as a potential conflict of interest.

## Publisher’s Note

All claims expressed in this article are solely those of the authors and do not necessarily represent those of their affiliated organizations, or those of the publisher, the editors and the reviewers. Any product that may be evaluated in this article, or claim that may be made by its manufacturer, is not guaranteed or endorsed by the publisher.

## Footnotes

^1^All names are pseudonyms.
